# Predicting hospital and emergency department utilization among community-dwelling older adults: Statistical and machine learning approaches

**DOI:** 10.1371/journal.pone.0206662

**Published:** 2018-11-01

**Authors:** Aaron Jones, Andrew P. Costa, Angelina Pesevski, Paul D. McNicholas

**Affiliations:** 1 Department of Health Research Methods, Evidence, and Impact, McMaster University, Hamilton, Ontario, Canada; 2 Department of Medicine, McMaster University, Hamilton, Ontario, Canada; 3 School of Computational Science and Engineering, McMaster University Hamilton, Ontario, Canada; 4 Department of Mathematics and Statistics, McMaster University, Hamilton, Ontario, Canada; University of Rochester, UNITED STATES

## Abstract

**Objective:**

The objective of this study was to compare the performance of several commonly used machine learning methods to traditional statistical methods for predicting emergency department and hospital utilization among patients receiving publicly-funded home care services.

**Study design and setting:**

We conducted a population-based retrospective cohort study of publicly-funded home care recipients in the Hamilton-Niagara-Haldimand-Brant region of southern Ontario, Canada between 2014 and 2016. Gradient boosted trees, neural networks, and random forests were tested against two variations of logistic regression for predicting three outcomes related to emergency department and hospital utilization within six months of a comprehensive home care clinical assessment. Models were trained on data from years 2014 and 2015 and tested on data from 2016. Performance was compared using logarithmic score, Brier score, AUC, and diagnostic accuracy measures.

**Results:**

Gradient boosted trees achieved the best performance on all three outcomes. Gradient boosted trees provided small but statistically significant performance gains over both traditional methods on all three outcomes, while neural networks significantly outperformed logistic regression on two of three outcomes. However, sensitivity and specificity gains from using gradient boosted trees over logistic regression were only in the range of 1%-2% at several classification thresholds.

**Conclusion:**

Gradient boosted trees and simple neural networks yielded small performance benefits over logistic regression for predicting emergency department and hospital utilization among patients receiving publicly-funded home care. However, the performance benefits were of negligible clinical importance.

## Introduction

Risk prediction models are commonly used across clinical practice for case-finding, triaging, and to inform clinical decision-making and care planning. Prognostic models have traditionally been derived using conventional statistical methods such as multivariable logistic regression. However, these classical approaches come with additivity and linearity assumptions which aid in the interpretability of the model but may represent first-order approximations of the true underlying relationships [1].

There are numerous algorithms from the machine learning and data mining literature that have been developed specifically for prediction. While these methods provide predictions only, rather than an interpretable model, they are considerably more flexible than traditional methods and can better account for non-linearities and interaction effects in predictors [2]. There has been considerable interest in recent years in using machine learning approaches to improve clinical risk prediction, with examples published in fields such as cardiology, rheumatology, oncology, and perioperative care [3–6]. However, studies comparing context-specific predictive performance of machine learning methods have yielded mixed results. While some studies have shown that machine learning methods offer significant performance improvements [7,8], some have found little difference [9], and others have concluded that traditional statistical approaches provide the best performance in some cases [10,11].

Hospital admission and emergency department (ED) utilization are of particular interest for case-finding among home and community care recipients as a key objective of community-based care is to shift patients away from hospital and institutional settings. The objective of this study was to compare the performance of several commonly used machine learning methods: neural networks, gradient boosted trees, and random forests, to two implementations of logistic regression for predicting ED and hospital utilization outcomes among patients receiving publicly-funded home care services. The aim was to determine if the implementation of machine learning methods in place of logistic regression would provide meaningful clinical benefits.

## Methods

### Study design and data sources

We conducted a population-based, retrospective cohort study of adult, home care recipients in the Hamilton-Niagara-Haldimand-Brant (HNHB) region of Ontario, Canada. The HNHB health region is one of the largest health regions in Ontario with a population of over 1.5 million persons spread across urban, suburban and rural locales. The health region contains over 10 municipalities with wide variations in population density, socioeconomic status, and access to care from tertiary centers.

We used multiple, linked, population-based health administrative databases to create the study cohort. Administrative and clinical home care records were obtained from the Client and Health Related Information System, which is the health administrative database used by Ontario’s publicly-funded home care system [12]. Information on ED visits and hospital stays were obtained from the National Ambulatory Care Reporting System and the Discharge Abstract Database, which contain standardized reporting for all ED visits and acute inpatient hospitalizations in Ontario [13,14]. The databases used in this study are regularly checked for validity and have been used extensively in research [15–17]. Anonymized regional versions of the administrative databases were accessed at the Health Data Library at McMaster University. We received ethics approval from the Hamilton Integrated Research Ethics Board (14-698-D) for secondary analysis of administrative health data, including waiver of informed consent.

### Participants

Community-dwelling adults receiving publicly-funded home and community care in Ontario are periodically assessed with the Resident Assessment Instrument: Home Care (RAI-HC), a comprehensive, standardized clinical assessment [18]. We constructed a population-based cohort of every patient 19 years of age or older that received a RAI-HC assessment in the HNHB region of Ontario between January 1^st^,2014 and December 31^st^,2016. The cohort was linked to ED records to identify any ED visits in the six months preceding and following the date of assessment, as well as the diagnoses (ICD-10-CA [19]) associated with each visit. The cohort was also linked to acute hospital inpatient records to identify admissions in the six months preceding and following the date of assessment.

### Measures

#### Outcomes

We examined a panel of three outcomes based on a patient’s usage of emergency and acute hospital inpatient services in the six months following the index RAI-HC assessment. The first outcome was any unplanned ED visit with both a fall and injury diagnosis code (S1 Table) within 182 days of the assessment date. The second outcome was any unplanned inpatient admission to an acute care hospital within 182 days of the index assessment date. The final outcome was a three-level categorical indicator of the number of unplanned ED visits in the 182 days following the RAI-HC assessment, with levels defined at 0 visits, 1 visit, or 2 or more visits.

#### Predictors

Predictors were taken from the index RAI-HC assessment as well as hospital and ED utilization patterns in the six months preceding the assessment. The RAI-HC is a comprehensive clinical assessment of over 250 items that has been found to be reliable and valid in documenting the domains of function, health, social support, and service use [20]. The internationally-developed assessment was created to identify multidimensional needs, aid in the development of appropriate care plans, and establish outcomes for tracking purposes. The RAI-HC has demonstrated good inter-assessor reliability and convergent validity with external gold standards [21–23]. RAI-HC assessment data have been used extensively in clinical and epidemiological research and the assessment is used in most Canadian provinces and territories, many U.S. states, and numerous countries in Europe and Asia/Pacific Rim [24,25].

We extracted all elements of the RAI-HC except for patient identifiers and a few items pertaining to the assessment itself (e.g., assessment date) or its associated community care referral (e.g., reason for referral) for use as predictors. We additionally extracted various clinical severity and risk scales and indicators that are secondarily derived from RAI-HC elements and are part of the traditional implementation of the assessment suite. These include validated measures of impairment in domains such as function, cognition, communication, health stability, and mood, and scales stratifying patients by risk of adverse outcomes. The number of unplanned ED visits, number of unplanned hospital admissions, and whether the patient had an ED visit with an injurious fall in the six months preceding the assessment date were also extracted from the hospital and ED records to serve as predictors.

### Analysis

#### Data segmentation

We segmented our cohort into separate training and test sets based on the year of assessment. Assessments from 2016 were reserved for final performance testing to mimic a realistic scenario in which a predictive model to be used with future data would be trained on past data. To train the predictive models, three distinct methods were implemented using the data from 2014–2015. First, models were trained using only data from 2015 and five-fold cross-validation within the 2015 data was used for model validation. The second set of models were trained similarly but used data from both years 2014 and 2015. The final set of models were also trained using only data from 2015. However, model validation was conducted using 2014 data to mimic the process of using one year’s data to predict the next year’s. The use of multiple training methods allows for the selection of the best training method for a given predictive method and enables a general comparison of differences in performance across the training methods.

#### Data preparation

The substantial breadth of the RAI-HC assessment elements makes it likely that a considerable proportion of elements have minimal or no predictive value for a given outcome. As the inclusion of such predictors comes with significant computational cost and can reduce performance, we implemented a variable selection step to reduce the number of predictors used for each outcome [26]. First, we created a Pearson correlation matrix between all elements in the full set of predictors and removed one predictor from any pairs that exhibited a correlation greater than 0.9. Next, the remaining predictors were scored using the mean decrease in accuracy variable importance measure from a conditional inference forest, which is a tree-based method that provides measures of unbiased variable importance [27]. We excluded all predictors below a mean decrease in accuracy threshold of 0.01%, separately for each outcome. As the purpose of this study is to compare the relative performance of predictive methods given a shared data set, the set of predictors for a given outcome is not required to be optimal but merely reasonable, an assumption that we examined via sensitivity analyses. Initial data extraction was performed with SAS 9.4 while data preparation and analysis were conducted using R 3.4.1[28].

### Predictive methods

#### Null model

A null, or intercept-only, model was used to provide a reference point to a model with no predictors. The null model assigns probabilities to outcomes equal to observed proportions and was fit using the mlr package.

#### Logistic regression

Logistic regression is the traditional method that models the log odds of a binary response as a linear combination of the predictor variables. While conventional logistic regression approaches often involve the selection of predictors based on expert knowledge or p-value thresholds, the models used in this study were not determined by a model building process but included all predictors irrespective of statistical significance or theoretical relevance. The logistic regression models were fit using the stats package.

#### Forward-stepping logistic regression with interactions and squared terms

A common practice to relax the additivity and linearity constraints of logistic regression is to include interactions between predictors and polynomial functions of predictors as separate variables in the model [29]. To represent this traditional method of increasing the flexibility of logistic regression, we developed a forward-stepping logistic regression function to consider two-way interactions and squared terms. The large number of predictors in this study rendered other methods such as best subsets or backwards regression computationally infeasible. Entry into the model was determined by the largest decrease in Akaike information criterion and followed a hierarchy in which interactions were only considered after the main effects entered. The forward-stepping logistic regression function was built utilizing the stats package.

#### Multinomial logistic regression

Logistic regression can be straightforwardly extended to dependent variables with more than two levels by choosing one level as a reference and fitting separate logistic models for every other level against the reference [30]. Multinomial logistic regression was fit using the nnet package.

#### Forward-stepping multinomial logistic regression with interactions and squared terms

The forward-stepping procedure to consider two-way interactions and squared terms previously described for binary logistic regression can be extended to multinomial logistic regression. This function was created utilizing the stats and nnet packages.

#### Neural networks

Network networks are non-linear regression and classification models represented as a network of layered nodes [1]. We used single-hidden layer networks with a logistic activation function and cross-entropy loss function. A weight decay parameter, analogous to shrinkage in ridge regression, was tuned to control overfitting. The size of the hidden layer and the weight decay parameter were tuned using a grid search. All inputs were normalized to have a mean of zero and standard deviation of 1. Neural networks were fit using the nnet package.

#### Gradient boosted trees

Gradient tree boosting builds an additive ensemble of regression trees by iteratively fitting new trees to the negative gradient of a loss function [31]. After an initial tree is trained on the data, successive trees are fit to the negative gradient of the ensemble up to that point. We tuned parameters controlling the maximum depth, minimum size of terminal nodes, and pruning threshold of the individual trees. Up to 1,000 rounds of boosting with trees up to a depth of 16 were considered. We also tuned a learning rate parameter to control overfitting, and a proportion of predictors and observations that are ignored each time individual tree is trained. Gradient tree boosting was performed using the xgboost package with parameters tuned using a random search.

#### Random forests

A random forest is an ensemble of classification trees that averages predictions over numerous trees built on bootstrap samples of the data [32]. Each split in each tree of a random forest only considers a random subset of the total predictors as candidates. The resulting trees exhibit low correlation with each other, allowing for an arbitrarily large number of trees to be built to reduce the variance of predictions. We built forests using the Gini index as the split criteria. The number of predictors considered in each tree and the minimum size of each node was tuned using a grid search. Following recent research, we did not tune the number of trees in the forest but set it to a large but computationally feasible number of 1,500 [33]. Random forests were fit with the ranger package.

### Performance measures

#### Logarithmic score

The logarithmic score is defined as $\frac{1}{N}\sum_{i=1}^{N}\ln{\hat{P}}_{i}$, where ${\hat{P}}_{i}$ is the predicted probability of the true class of the *i*th observation. The logarithmic score is a strictly proper scoring rule, meaning it is maximized only by probabilities drawn from the true probability distribution [34]. It is equivalent to averaging the contributions of individual observations to the binomial or multinomial log-likelihood function.

#### Brier score

The Brier score is another strictly proper scoring rule that is defined as $\frac{1}{N}\sum_{i=1}^{N}\sum_{j=1}^{C}{\left({\hat{P}}_{ij}-Y_{ij}\right)}^{2}$, where ${\hat{P}}_{ij}$ is the predicted probability of the *j*th class in the *i*th observation and *Y_ij_* is 1 when the true class of the observation is *j* and 0 otherwise [35]. In contrast to the other two performance measures, lower Brier scores represent better predictive performance.

#### AUC

The area under the receiver operating curve (AUC), or c-statistic, is the area under the curve created by plotting sensitivity against 1-specificity at various thresholds. The AUC is a common measure of the discriminative ability of a risk models but is invariant to the calibration of predicted probabilities. For the multinomial outcome, the AUC was reported as the average of the AUC of each level against the other levels, weighted by the observed proportion of each level [36].

#### Diagnostic accuracy measures

As none of the probabilistic performance measures lend themselves to straightforward clinical interpretation, traditional diagnostic accuracy measures were produced for the two binary outcomes to better judge the clinical importance of performance differences. Two separate thresholds were explored, the first fixing the sensitivity at 80% and the second setting the threshold at the 80^th^ percentile of the predicted probability distribution of the testing data. Sensitivity, specificity, positive and negative likelihood ratios, and the diagnostic odds ratio were reported.

#### Performance testing

Performance was measured by calculating the performance metrics on predictions for the previously unseen calendar year 2016 assessment data. Statistical analysis was performed using pairwise paired t-tests of the difference in logarithmic score with Hochberg’s correction for multiple comparisons. The statistical analysis and the assessment of the clinical importance of predictive differences was performed using results from the best training method for a given outcome and predictive method. ROC curves were drawn for the binary outcomes using the best performing training method.

### Sensitivity analysis

We assessed the impact of our exclusion of predictors based on the conditional inference forest variable importance measure by comparing the logarithmic score obtained using the full set of predictors to the logarithmic score produced using the reduced set that was utilized in the main analysis. Comparisons were made for logistic regression and random forests with default parameter settings using the training method that only considered 2015 data.

## Results

We identified 88,364 RAI-HC assessed cases between January 1^st^, 2014 and December 31^st^, 2016 for inclusion in the study. There were 19 cases (0.02%) with missing data that were removed from the analysis, reducing the final number to 88,345. The number of cases increased slightly each year: 29,132 in 2014, 29,278 in 2015, and 29,935 in 2016. A descriptive clinical profile of the community care patients in the study can be found in Table 1. Patients tended to be elderly with a median age of 82 years. A majority of patients were female (62.3%). Impairments in function (66.7%) and cognition (67.4%) were common, as were cardiovascular (52.4%) and musculoskeletal disease (66.3%).

**Table 1:** Characteristics of study participants.

Year of Assessment	2014 (n = 29,132)	2015 (n = 29,278)	2016 (n = 29,935)
	n(%)	n(%)	n(%)
**Demographic Characteristics**			
Age (Median (IQR))	82 (16)	82 (16)	82 (16)
Sex (F)	18,497 (63.49)	18,301 (62.51)	18,250 (60.97)
Lives Alone	10,169 (34.91)	9,985 (34.10)	10,050 (33.57)
**Health Characteristics**			
ADL Impairment^a^	18,489 (63.47)	19,423 (66.34)	21,004 (70.17)
Cognitive Impairment^b^	18,789 (64.50)	19,776 (67.55)	20,958 (70.01)
Fall in last 90 days	12,104 (41.55)	13,051 (44.58)	14,181 (47.37)
Bladder Incontinence	12,393 (42.54)	12,728 (43.47)	13,315 (44.48)
Poor Self-Reported Health	5,956 (20.44)	6,253 (21.36)	8,173 (27.30)
Dyspnea	8,191 (28.12)	8,492 (29.00)	9,047 (30.22)
Mood Symptoms^c^	12,843 (44.09)	13,644 (46.60)	15,239 (50.91)
Aggressive Behaviour^d^	2,659 (9.13)	3,057 (10.44)	3,650 (12.19)
Wandering	803 (2.76)	9,37 (3.20)	1,101 (3.68)
Number of Medications (Mean (SD))	7.40 (2.33)	7.41 (2.32)	7.37 (2.34)
**Informal Caregiver Status**			
Caregiver expresses distress^e^	6,093 (20.92)	6,743 (23.03)	9,507 (31.76)
Informal care hours per day (Mean (SD))	19.57 (22.11)	19.47 (21.87)	20.32 (23.92)
**Diagnoses**			
Cardiovascular^f^	15,053 (51.67)	15,301 (52.26)	15,958 (53.31)
Dementia	6,869 (23.58)	7,280 (24.87)	7,915 (26.44)
Neurological^g^	2,981 (10.23)	3,341 (11.41)	3,961 (13.23)
Musculoskeletal^h^	19,141 (65.70)	19,389 (66.22)	20,028 (66.90)
Psychiatric^i^	5,995 (20.58)	6,408 (21.89)	7,183 (24.00)
Cancer	3,669 (12.59)	3,779 (12.91)	4,014 (13.41)
Diabetes	7,700 (26.43)	7,754 (26.48)	8,316 (27.78)
COPD	5,464 (18.76)	5,581 (19.06)	5,793 (19.35)

^a^ADL Long Form > 0.

^b^Cognitive Performance Scale > 0.

^c^Depression Rating Scale > 0.

^d^Verbal abuse, physical abuse, socially inappropriate behavior, or resistance to care in last 3 days.

^e^Caregiver expresses feelings of distress, anger, or depression.

^f^Stroke, congestive heart failure, coronary artery disease, dysrhythmia, peripheral vascular disease.

^g^Head trauma, hemiplegia, multiple sclerosis, parkinsonism.

^h^Arthritis, fracture, osteoporosis.

^i^ Any psychiatric diagnosis

The proportion of patients that visited the ED with an injurious fall within 6 months of the index assessment was 9.1% in 2014, 9.8% in 2015, and 9.7% in 2016 (Table 2). The proportion of patients who had an unplanned inpatient admission to an acute care hospital was 27.4% in 2014, and 28.0% in both 2015 and 2016. Slightly over half of the patients (53.4%, 52.5%, and 51.6%) did not visit the ED in the 6-month window across all three years, while roughly one quarter went once (24.6%) and the remaining (22.0%, 22.9% and 23.8%) went twice or more.

**Table 2:** Distribution of observed outcomes.

Year of Assessment	2014	2015	2016
Outcome	%	%	%
ED visit with injurious fall	9.1	9.8	9.7
Unplanned hospital admission	27.4	28.0	28.0
ED visit count—0	53.4	52.5	51.6
ED visit count—1	24.6	24.6	24.6
ED visit count—2+	22.0	22.9	23.8

A total of 319 potential predictors were extracted from the RAI-HC. We eliminated 29 predictors due to correlations greater than 0.9 with other predictors. Applying the 0.01% variable importance threshold to the assessment data from 2015 reduced the final number of predictors to 113, 120, and 108 for the ED visit with injurious fall, hospital admission, and ED visit count outcomes respectively. When training on data from both 2014 and 2015 the variable importance threshold dropped the final number of predictors to 94, 96, and 71. A list of the predictors included for each outcome can be found in S2–S4 Tables.

### ED visit with an injurious fall

Gradient boosted trees achieved the best overall performance on the ED visit with an injurious fall outcome, achieving a maximum logarithmic score of -0.301 and AUC of 0.679, which occurred when data from 2014 was used to validate models then trained on 2015 (Table 3). Within the other two training methods, neural networks and gradient boosted trees were tied for the best performance. Across the training methods, both neural networks and gradient boosted trees were superior to both logistic regression and the forward-stepping logistic regression function.

**Table 3:** Performance metrics for the ED with injurious fall outcome.

Outcome	Training Method	Score	Method					
			Null	LR	FL	NN	GBT	RF
ED visit with injurious fall	2015-training	Logarithmic	-0.319	-0.305	-0.306	-0.303	-0.303	-0.306
		Brier	0.176	0.171	0.170	0.170	0.170	0.171
		AUC	0.500	0.663	0.663	0.668	0.668	0.659
							
	2014-training 2015-training	Logarithmic	-0.319	-0.303	-0.303	-0.302	-0.302	-0.304
		Brier	0.176	0.170	0.170	0.170	0.169	0.170
		AUC	0.500	0.670	0.671	0.673	0.673	0.664
							
	2014-validation 2015-training	Logarithmic	-0.319	-0.305	-0.306	-0.303	**-0.301**	-0.305
		Brier	0.176	0.171	0.170	0.170	**0.169**	0.170
		AUC	0.500	0.663	0.666	0.668	**0.679**	0.659

LR, logistic regression; FL, Forward-stepping logistic regression with interactions and squared terms; NN, Neural Network; GBT, Gradient boosted trees; RF, Random forest.

### Unplanned hospital admission

Gradient boosted trees also achieved the best overall performance on the unplanned hospital admission outcome, with a maximum logarithmic score -0.545 and an AUC of 0.689 occurring when both 2014 and 2015 were used as training data (Table 4). Gradient boosted trees attained the highest performance across all the training methods, and once again both neural networks and gradient boosted trees were always superior to both traditional methods.

**Table 4:** Performance metrics for the unplanned hospital admission outcome.

Outcome	Training Method	Score	Method					
			Null	LR	FL	NN	GBT	RF
Unplanned hospital admission	2015-training	Logarithmic	-0.593	-0.554	-0.553	-0.551	-0.547	-0.552
		Brier	0.404	0.372	0.372	0.369	0.367	0.370
		AUC	0.500	0.675	0.675	0.680	0.686	0.683
	2014-training 2015-training	Logarithmic	-0.593	-0.551	-0.550	-0.548	**-0.545**	-0.551
		Brier	0.404	0.369	0.369	0.367	**0.365**	0.369
		AUC	0.500	0.681	0.681	0.685	**0.689**	0.686
	2014-validation 2015-training	Logarithmic	-0.593	-0.554	-0.553	-0.551	-0.546	-0.551
		Brier	0.404	0.372	0.372	0.369	0.366	0.369
		AUC	0.500	0.675	0.675	0.680	0.688	0.683

LR, logistic regression; FL, Forward-stepping logistic regression with interactions and squared terms; NN, Neural Network; GBT, Gradient boosted trees; RF, Random forest.

### ED visit count

The best performance on the ED visit count outcome was also attained by gradient boosted trees, with a maximum logarithmic score of -0.962 and AUC of 0.655 occurring when data from 2014 was used to validate models then trained on 2015 (Table 5). Similar to the previous outcomes, gradient boosted trees achieved the highest score across all the methods, though it was tied with neural networks on the method that used both 2014 and 2015 data for training.

**Table 5:** Performance metrics for the ED visit count outcome.

Outcome	Training Method	Score	Method					
			Null	MLR	MFL	NN	GBT	RF
ED visit count	2015-training	Logarithmic	-1.029	-0.972	-0.972	-0.968	-0.965	-0.969
		Brier	0.617	0.577	0.577	0.575	0.573	0.576
		AUC	0.500	0.643	0.643	0.646	0.655	0.651
	2014-training 2015-training	Logarithmic	-1.029	-0.968	-0.968	-0.965	-0.965	-0.969
		Brier	0.617	0.575	0.575	0.573	0.573	0.575
		AUC	0.500	0.648	0.647	0.651	0.655	0.652
	2014-validation 2015-training	Logarithmic	-1.029	-0.972	-0.972	-0.967	**-0.962**	-0.968
		Brier	0.617	0.577	0.577	0.575	**0.571**	0.575
		AUC	0.500	0.643	0.643	0.647	**0.655**	0.651

MLR, multinomial logistic regression; MFL, Forward-stepping multinomial logistic regression with interactions and squared terms; NN, Neural Network; GBT, Gradient boosted trees; RF, Random forest.

### Statistical analysis

The results of the pairwise paired t-tests of difference in logarithmic score with Hochberg’s correction can be seen in Table 6. Notably, the performance of gradient boosted trees was significantly different from both traditional methods for each outcome, while the performance of random forests was never statistically different from either conventional method. The neural network was significantly different from logistic regression on the unplanned hospital admission and ED visit count outcomes.

**Table 6:** Pairwise paired t-tests of difference in logarithmic score with Hochberg’s correction.

Outcome	Method	LR	FL	NN	GBT
ED visit with injurious fall	**FL**	0.700			
	**NN**	0.068	0.700		
	**GBT**	0.011	0.035	0.110	
	**RF**	0.110	0.106	0.017	<0.001
		**LR**	**FL**	**NN**	**GBT**
Unplanned hospital admission	**FL**	0.279			
	**NN**	<0.001	0.099		
	**GBT**	<0.001	<0.001	0.009	
	**RF**	0.988	0.426	0.007	<0.001
		**LR**	**FL**	**NN**	**GBT**
ED visit count	**FL**	0.949			
	**NN**	<0.001	<0.001		
	**GBT**	<0.001	<0.001	0.008	
	**RF**	0.949	0.949	0.008	<0.001

LR, logistic regression; FL, Forward-stepping logistic regression with interactions and squared terms; NN, Neural Network; GBT, Gradient boosted trees; RF, Random forest.

ROC curves for the two binary outcomes created using the best training method for each predictive method can be found in S1 Fig and S2 Fig. There was little discernable difference in ROC curves between any of the predictive methods.

### Clinical importance

Differences in the diagnostic accuracy measures between the predictions produced by gradient boosted trees and the traditional statistical methods were small across all outcomes and thresholds. For the ED visit with injurious fall outcome, fixing the sensitivity at 80% resulted in the gradient boosted tree ensemble attaining a specificity of 42.9%, compared to 41.8% and 41.2% for the logistic regression and the forward-stepping logistic regression methods respectively (Table 7). Fixing the threshold at the 80^th^ percentile of the 2016 predicted probability distribution resulted in the gradient boosted tree ensemble attaining a sensitivity of 38.9%, with logistic regression attaining 37.5% and forward-stepping logistic method also attaining 38.9%.

**Table 7:** Diagnostic accuracy measures for the ED visit with injurious fall outcome.

Threshold	Measure	Method				
		LR	FL	NN	GBT	RF
Sensitivity fixed at 80%	Sensitivity	80.1%	80.1%	80.1%	**80.2%**	80.2%
	Specificity	41.8%	41.2%	42.6%	**42.9%**	40.0%
	LR+	1.38	1.36	1.39	**1.40**	1.34
	LR-	0.48	0.48	0.47	**0.46**	0.50
	Odds Ratio	2.89	2.82	2.98	**3.04**	2.70
80% of predicted probability distribution						
	Sensitivity	37.5%	**38.9%**	38.6%	**38.9%**	36.5%
	Specificity	81.9%	**82.0%**	82.0%	**82.0%**	82.0%
	LR+	2.07	**2.17**	2.14	**2.16**	2.03
	LR-	0.76	**0.74**	0.75	**0.75**	0.77
	Odds Ratio	2.72	**2.91**	2.86	**2.91**	2.61

LR, logistic regression; FL, Forward-stepping logistic regression with interactions and squared terms; NN, Neural Network; GBT, Gradient boosted trees; RF, Random forest.

Fixing the sensitivity at 80% for the unplanned hospital admission outcome resulted in gradient boosted trees attaining a specificity of 43.8%, compared to 42.1% and 41.8% for the logistic regression methods (Table 8). Fixing the threshold at the 80^th^ percentile of the 2016 predicted probability distribution resulted in gradient boosted trees attaining a sensitivity of 35.6% compared to 34.7% and 34.4% for the traditional methods.

**Table 8:** Diagnostic accuracy measures for the unplanned hospital admission outcome.

Threshold	Measure	Method				
		LR	FL	NN	GBT	RF
Sensitivity fixed at 80%	Sensitivity	80.0%	80.1%	79.9%	**80.0%**	80.0%
	Specificity	42.1%	41.8%	42.7%	**43.8%**	42.7%
	LR+	1.38	1.38	1.40	**1.42**	1.40
	LR-	0.47	0.48	0.47	**0.46**	0.47
	Odds Ratio	2.91	2.89	2.97	**3.11**	2.99
80% of predicted probability distribution						
	Sensitivity	34.7%	34.4%	35.3%	**35.6%**	35.5%
	Specificity	85.7%	85.6%	86.0%	**86.1%**	86.0%
	LR+	2.43	2.39	2.52	**2.55**	2.53
	LR-	0.76	0.77	0.75	**0.75**	0.75
	Odds Ratio	3.19	3.13	3.35	**3.41**	3.37

LR, logistic regression; FL, Forward-stepping logistic regression with interactions and squared terms; NN, Neural Network; GBT, Gradient boosted trees; RF, Random forest.

### Sensitivity analysis

The predictive performance of both logistic regression and random forests was worse using the full predictor set than using the reduced set for each outcome, although differences were small (S5 Table).

## Discussion

This study compared the performance of several commonly used machine learning methods to traditional statistical methods for predicting the probability of ED and hospitalization utilization outcomes among patients receiving community-based care. Gradient tree boosting was the best performing method, achieving the top predictive performance on all three outcomes, although the differences in performance were small. Neural networks also outperformed the logistic regression methods across all outcomes and was tied with gradient boosted trees in several instances. Neither random forests nor logistic regression with interactions and squared terms provided a statistically significant improvement over basic logistic regression for any outcome.

As the statistical significance of predictive differences does not indicate their clinical utility, we also sought to determine the clinical importance of the performance gains. We examined the clinical importance of the predictive differences by comparing various diagnostic accuracy measures for the two binary outcomes at two classification thresholds. The first threshold was set to produce a posited minimum acceptable sensitivity of 80% while the second threshold was set at the 80^th^ percentile of the predicted probability distribution of the 2016 assessment population, emulating a resource-constrained case-finding scenario in which the top 20% of patients at risk would be eligible for an intervention.

Across the outcomes and thresholds examined, differences in diagnostic accuracy measures from predictive performance increases were minimal. When the sensitivity was fixed at 80%, gradient tree boosting achieved specificity gains of 1%-2% over the traditional statistical methods for both the ED visit with injurious falls and unplanned hospital admission outcomes. In the case-finding scenario, gradient tree boosting achieved sensitivity increases of around 1% over the traditional methods for the two outcomes examined, although it offered no improvement over the more flexible logistic regression method for the ED visit with injurious fall outcome. Absolute gains were similarly small. Replacing a logistic regression model with a gradient boosted tree ensemble for identifying the top 20% of patients at risk in a case-finding application would result in capturing 1.3 more true positives per 1,000 patients screened for the falls outcome and 2.4 more true positives per 1,000 patients for the unplanned hospital admission outcome.

Although this study found no clinical benefit in replacing logistic regression with machine learning methods, the relative performance of predictive methods for a given problem will depend on the importance and complexity of non-linear and non-additive relationships within predictors. Other areas within community-based care, such as in-home monitoring, may offer data sources of higher dimensionality and complexity that would benefit more from machine learning approaches. Furthermore, there are a myriad of machine learning methods available, and others not examined in this study may have greater success. In particular, we note that the single-layer, feed-forward neural networks that we examined in this study are among the simplest of networks, but they consistently achieved good relative performance. Neural networks with multiple hidden layers, convolution networks, or other deep learning approaches may be able to perform better. Also, the logistic regression methods utilized in this study, which included all available predictors irrespective of statistical significance or theoretical relevance, may have performed better than some of the common expert-guided or p-value driven model building approaches.

This study has a few notable limitations. First, although the generalizability of our results is aided by the examination of several important outcomes and the widespread use of the RAI-HC and related assessments internationally, our findings may not apply outside of a community care context. In addition, the complexity of the factors that impact emergency department and hospital utilization may vary by jurisdiction, which could impact the relative ranking of the predictive methods.

## Conclusion

We compared the performance of several commonly used machine learning methods against two logistic regression methods for predicting the utilization of hospital and emergency services among patients receiving home care services. We found that gradient boosted trees and simple neural networks provided small performance improvements over traditional methods. However, the clinical importance of the differences in performance was negligible. While this study found no clinical benefit in replacing logistic regression with gradient boosted trees or simple neural networks, researchers should continue to examine methods emerging from the machine learning and data mining fields to determine to what degree they can be employed to improve clinical practice.

## Supporting information

S1 Table List of ICD-10-CA codes used to define falls and injuries. (DOCX)

S2 Table Predictors used in the ED visit with injurious fall outcome by descending importance. (CSV)

S3 Table Predictors used in the unplanned hospital admission outcome by descending importance. (CSV)

S4 Table Predictors used in the ED visit count outcome by descending importance. (CSV)

S5 Table Sensitivity analysis of predictive performance to use of variable selection. (DOCX)

S1 Fig ROC curves for the ED visit with injurious fall outcome. (TIF)

S2 Fig ROC curves for the unplanned hospital admission outcome. (TIF)
