# Supplementary material for: Predicting hospital and emergency department utilization among community-dwelling older adults: Statistical and machine learning approaches
Source: PLoS One. 2018 Nov 1;13(11):e0206662. doi: 10.1371/journal.pone.0206662 (PMC6211724; doi:10.1371/journal.pone.0206662)
Supplement: S1 Table — (DOCX) [file pone.0206662.s001.docx]

| **Code Type** | **ICD-10-CA** |
| --- | --- |
|  |  |
| Fall codes | W00-W19 |
|  |  |
| Injury codes | S00-S99 |
|  | T00-T14 |
|  | T20-T32 |
|  | W20-W99 |
