# Supplementary material for: Predicting hospital and emergency department utilization among community-dwelling older adults: Statistical and machine learning approaches
Source: PLoS One. 2018 Nov 1;13(11):e0206662. doi: 10.1371/journal.pone.0206662 (PMC6211724; doi:10.1371/journal.pone.0206662)
Supplement: S5 Table — (DOCX) [file pone.0206662.s005.docx]

| **Outcome** | **Method** | **Logarithmic Score** | |
| --- | --- | --- | --- |
|  |  | **Reduced Predictors** | **All Predictors** |
| ED visit with injurious fall | Logistic Regression | -0.305 | -0.309 |
|  | Random Forest | -0.306 | -0.308 |
| Unplanned hospital admission | Logistic Regression | -0.554 | -0.556 |
|  | Random Forest | -0.552 | -0.553 |
| ED visit count | Logistic Regression | -0.972 | -0.981 |
|  | Random Forest | -0.971 | -0.972 |
