# Supplementary figures and images for: Predicting hospital and emergency department utilization among community-dwelling older adults: Statistical and machine learning approaches
Source: PLoS One. 2018 Nov 1;13(11):e0206662. doi: 10.1371/journal.pone.0206662 (PMC6211724; doi:10.1371/journal.pone.0206662)

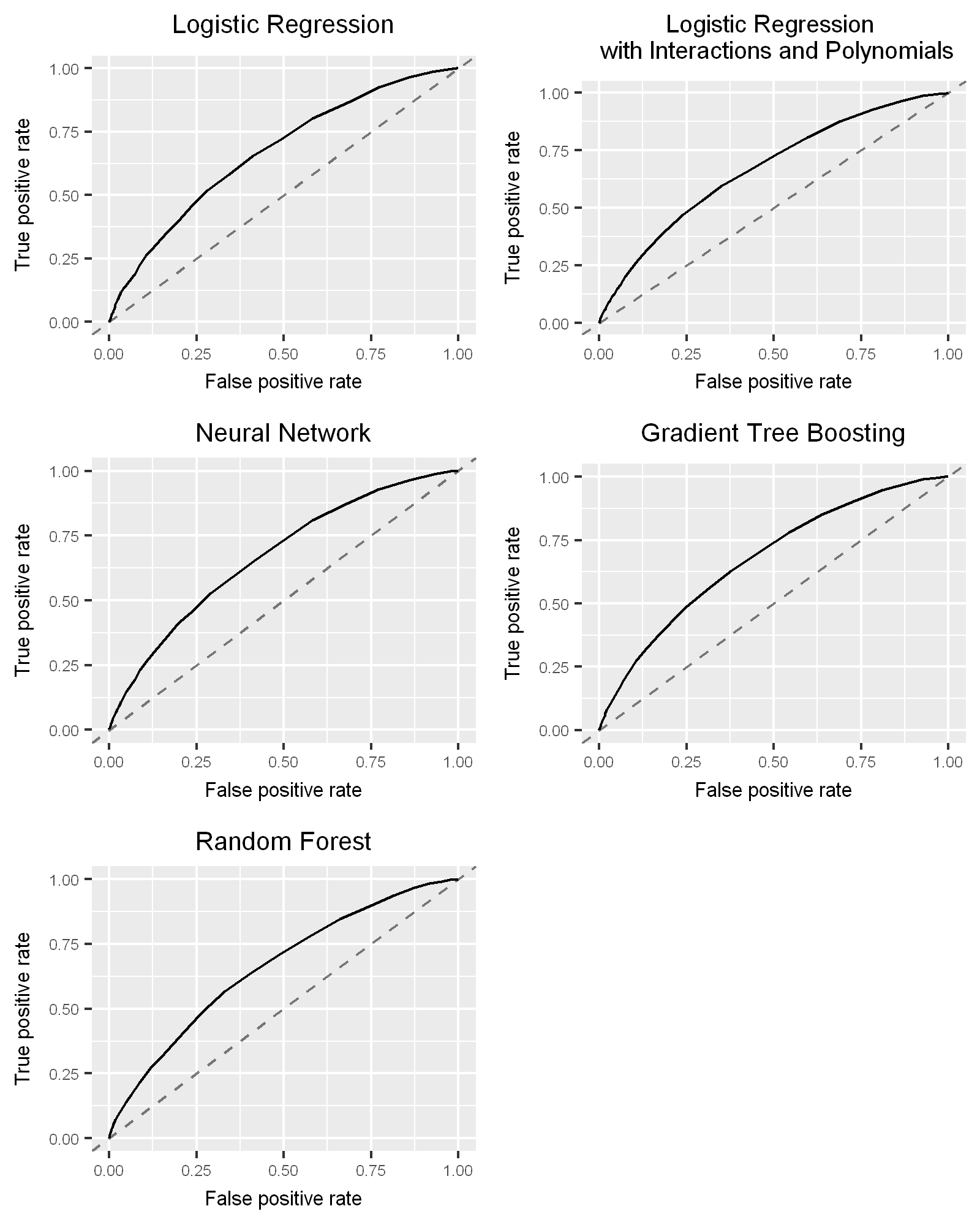

Supplement: S1 Fig — (TIF) [file pone.0206662.s006.tif]

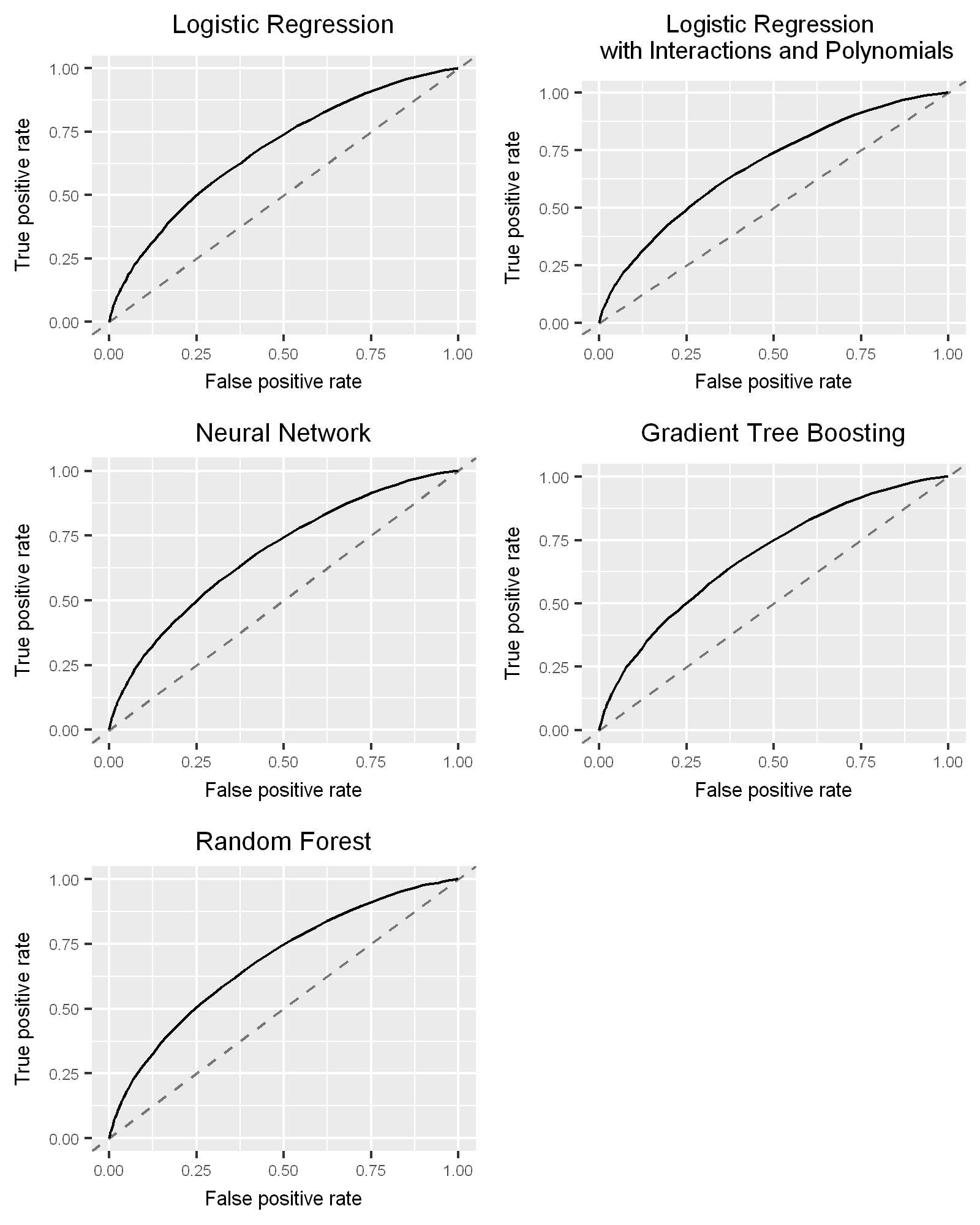

Supplement: S2 Fig — (TIF) [file pone.0206662.s007.tif]
